# *Burkholderia cenocepacia* conditional growth mutant library created by random promoter replacement of essential genes

**DOI:** 10.1002/mbo3.71

**Published:** 2013-02-07

**Authors:** Ruhi A M Bloodworth, April S Gislason, Silvia T Cardona

**Affiliations:** Department of Microbiology, University of ManitobaWinnipeg, Manitoba, Canada

**Keywords:** Burkholderia cepacia complex, chemical genetics, essential genome, growth inhibition, inducible gene expression, large scale bacterial genetics

## Abstract

Identification of essential genes by construction of conditional knockouts with inducible promoters allows the identification of essential genes and creation of conditional growth (CG) mutants that are then available as genetic tools for further studies. We used large-scale transposon delivery of the rhamnose-inducible promoter, P*rhaB* followed by robotic screening of rhamnose-dependent growth to construct a genomic library of 106 *Burkholderia cenocepacia* CG mutants. Transposon insertions were found where P*rhaB* was in the same orientation of widely conserved, well-characterized essential genes as well as genes with no previous records of essentiality in other microorganisms. Using previously reported global gene-expression analyses, we demonstrate that P*rhaB* can achieve the wide dynamic range of expression levels required for essential genes when the promoter is delivered randomly and mutants with rhamnose-dependent growth are selected. We also show specific detection of the target of an antibiotic, novobiocin, by enhanced sensitivity of the corresponding CG mutant (P*rhaB* controlling *gyrB* expression) within the library. Modulation of gene expression to achieve 30–60% of wild-type growth created conditions for specific hypersensitivity demonstrating the value of the CG mutant library for chemogenomic experiments. In summary, CG mutants can be obtained on a large scale by random delivery of a tightly regulated inducible promoter into the bacterial chromosome followed by a simple screening for the CG phenotype, without previous information on gene essentiality.

## Introduction

The *Burkholderia cepacia* complex (referred to here as Bcc) is a group of closely related gram-negative bacteria that are widely distributed in natural and man-made environments (Vandamme and Dawyndt 2011). *Burkholderia* species, including the Bcc, are of great interest because of their large multipartite genomes, their great metabolic versatility, and the wide array of ecological niches they occupy (Sousa et al. 2011). Also of interest is the “dual personality” of the Bcc as these environmental bacteria, which were initially considered harmless, are now known to cause human infections (Chiarini et al. 2006). While strains can be exploited for biocontrol, bioremediation, and plant growth promotion purposes, safety issues arise regarding human infections, as many Bcc strains have emerged as antibiotic-resistant, opportunistic pathogens in patients with cystic fibrosis, chronic granulomatous disease, and other medical conditions associated with a compromised immune system (Mahenthiralingam et al. 2005; Valvano et al. 2005; Loutet and Valvano 2010).

Bacterial genes that are required for growth in rich, undefined media are regarded as essential and hence their encoded products are potential targets of new antibiotics (Brown and Wright 2005). Essential genes have been identified on a genomic scale by high-density transposon knockout mutagenesis (Hutchison et al. 1999; Akerley et al. 2002; Gerdes et al. 2003; Jacobs et al. 2003; Sassetti et al. 2003) or systematic gene-by-gene inactivation (Thanassi et al. 2002; Kang et al. 2004) where genes for which mutants could not be recovered are assumed to be essential. However, identification of essential genes by construction of conditional knockouts with inducible promoters adds the value of obtaining conditional growth (CG) mutants that are then available for further studies (Judson and Mekalanos 2000; DeVito et al. 2002; Forsyth et al. 2002). A number of inducible promoters have been used to express essential genes through the construction of CG mutants (Wong and Akerley 2003; Carroll et al. 2005), with the *Escherichia coli* arabinose-inducible promoter (PBAD, P*araB*) being one of the best characterized (Guzman et al. 1995; Judson and Mekalanos 2000). A great challenge is, however, to achieve genomic representation of essential genes with conditional mutagenesis, probably because of the different range of required expression levels. Promoters with very low uninduced expression levels are necessary to obtain mutants with a CG phenotype. Yet, highly induced expression levels may be necessary for highly expressed essential genes. Promoters that are inducible to such high levels may show uninduced levels of essential gene expression that are tolerable to bacterial growth (Bugrysheva et al. 2011).

The *E. coli* rhamnose-inducible promoter (P*rhaB*) is controlled by a cascade of two transcriptional regulators and is more tightly regulated than P*araB* (Haldimann et al. 1998). We previously demonstrated that P*rhaB* is suitable for tightly regulated gene expression in the Bcc clinical isolate *B. cenocepacia* K56-2, and that essential genes can be identified by transposon-based delivery of P*rhaB* throughout the bacterial chromosome followed by screening for absence or growth without rhamnose (Cardona et al. 2006).

In this work, we asked whether saturation of a genome with P*rhaB* allows identification of essential genes at the genomic level with representation of such genes in a library of CG mutants. Using a large-scale mutagenesis approach and robotic screening of more than 200,000 transposon mutants for rhamnose-dependent growth, we constructed a library of CG mutants (CG mutant library) and analyzed the contribution of promoter expression levels and gene redundancy in the identification of essential genes. We demonstrate that screening for CG in one condition identifies CG mutants of similar CG phenotypes, which makes them suitable for chemogenomic experiments.

## Experimental Procedures

### Bacterial strains and growth conditions

Bacterial strains and plasmids are listed in Table 1, and the identified CG mutants are listed in Table S1. All mutants were made in a *B. cenocepacia* K56-2 background and were grown in Luria Bertani (LB) media at 37°C, supplemented as required with different concentrations of rhamnose, 100 or 50 μg/mL trimethoprim (Tp) for *B. cenocepacia* or *E. coli*, respectively, 50 μg/mL gentamicin and 40 μg/mL kanamycin (Km). All chemicals were purchased from Sigma Chemical Co., St. Louis, MO unless otherwise indicated. To prepare standardized glycerol stocks, overnight cultures were washed twice with LB, adjusted to a final OD_600nm_ of 0.2 in LB 20% glycerol, and aliquoted into polymerase chain reaction (PCR) tubes for storage at −70°C. In assays involving bacterial growth, cultures were diluted to give a theoretical final OD_600nm_ of 0.001, and arranged in 96-well format. Plates were sealed with parafilm and incubated at 37°C with shaking at 200 rpm in a New Brunswick Scientific E24 shaking incubator (Edison, NJ). OD_600nm_ readings were taken using BioTek Synergy 2 plate reader (Winooski, VT).

**Table 1:** Bacterial strains and plasmids

	Features	Source
Strains		
*Burkholderia cenocepacia* K56-2 (LMG18863)	ET12 linage, CF isolate	Mahenthiralingam et al. (2000)
*B. cenocepacia gyrB*	Promoter replacement of *gyrB* P*gyrB*:: pRB6	This study
*Escherichia coli* SY327	*araD* Δ(*lac pro*) *argE* (Am) *recA*56 Rif^r^ *nalA λ pir*	Miller and Mekalanos (1988)
*E. coli* DH5*α*	F^−^, ϕ 80 *lac*ZΔM15 *endA1 recA1 hsdR*17(r_K_^−^m_K_^+^)*supE*44 *thi−*1 Δ*gyrA*96 (*ΔlacZYA*-*arg*F)U169 *relA*1	Invitrogen
Plasmids		
pRK2013	*ori*_colE1_, RK2 derivative, Km^r^ *mob*^+^ *tra*^+^	Figurski and Helinski (1979)
pRB-rham	pSCrhaboutgfp derivative (Cardona et al. 2006), *ori*_R6K_, *rhaR rhaS PrhaB* e-*gfp*	This study
pSC200	pGpΩTp derivative (Flannagan et al. 2007), *ori*_R6K_, *rhaR rhaS PrhaB* e-*gfp*	Ortega et al. (2007)
pRB6	pSC200 containing *gyrB* 5′end	This study

### Molecular biology techniques

DNA ligase and restriction enzymes (New England Biolabs, Whitby, ON, Canada) were used as recommended by the manufacturers. *Escherichia coli* SY327 cells were transformed using the Z-competent buffer kit protocol (Zymo Research, Irvine, CA). Conjugation into *B. cenocepacia* K56-2 was accomplished by triparental mating (Craig et al. 1989) with *E. coli* DH5*α* carrying the helper plasmid pRK2013 (Figurski and Helinski 1979). DNA was amplified using a PTC-221 DNA engine (MJ Research, Waltham, MA) or an Eppendorf Mastercycler ep gradient S thermal cycler with either Taq DNA polymerase (Qiagen, Hilden, Germany) or Phusion High-Fidelity PCR Kit (New England Biolabs). Amplification conditions were optimized for each primer pair. PCR products and plasmids were purified using QIAquick PCR Purification Kit (Qiagen) and QIAprep Spin Miniprep Kit (Qiagen), respectively. DNA sequencing was performed by The Center for Applied Genomics (TCAG) at The Hospital for Sick Children, Toronto, Ontario.

### Vector constructions

**Figure 1:**
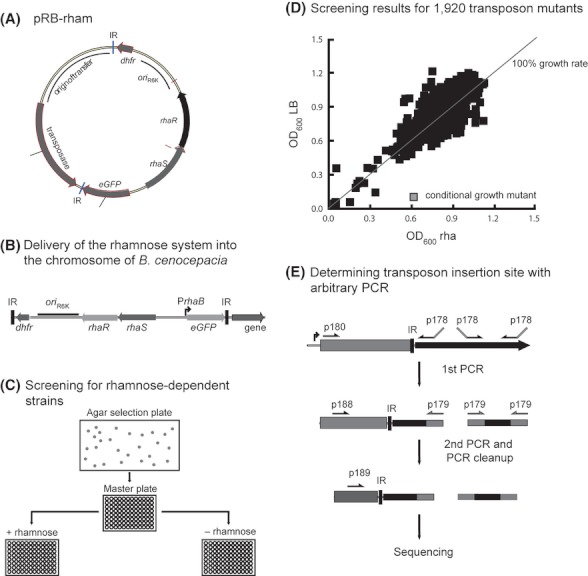
Construction of a *Burkholderia cenocepacia* CG mutant library. (A) Transposon vector pBR-rham is a derivative of pSCrhaBout*gfp* (Cardona et al. 2006). See Experimental Procedures for details on plasmid construction. (B) Trimethoprim resistance provided by the *dhfr* cassette was used to select for the transconjugants containing an outward-facing rhamnose-inducible promoter P*rhaB*. (C) The transconjugants were robotically picked into 96- or 384-well master plates before being robotically replicated into 96- or 384-well secondary plates containing LB with and without rhamnose. (D) The OD_600nm_ of the plates were read after 16 h and mutants showing at least 50% less growth in the absence of rhamnose were included in the library. (E) The insertion sites of the mutants were primarily determined using arbitrary primed PCR to preferentially amplify the transposon–genome junction and sequenced using a transposon-specific primer.

pRB-rham (Fig. 1A) is a derivative of pSCrhaBoutgfp (Cardona et al. 2006), in which the pMB1 origin of replication was replaced with that of the R6K plasmid (*ori*_R6K_) to avoid possible reversion to a replicative plasmid in *B. cenocepacia. ori*_R6K_-dependent replication has the absolute requirement of the Pir protein (Stalker et al. 1979), and thus plasmids bearing this replication origin do not replicate in bacteria not harboring *pir*.

To construct pRB-rham, pTnMod-RTp (Dennis and Zylstra 1998) and pSCrhaBoutgfp (Cardona et al. 2006) were digested with *SpeI/KpnI* and the *ori*_R6K_, and *dhfr* cassette from pTnMod-RTp was ligated to the backbone of pSCrhaBoutgfp. To construct plasmid pRB6, a 300-bp DNA fragment containing the 5′ end of *gyrB*, flanked by *XbaI* and *NdeI* restriction sites, was cloned into pSC200 immediately downstream from the rhamnose-inducible promoter. The resulting plasmid was conjugated into *B. cenocepacia* K56-2 by triparental mating. Integration of pRB6 and replacement of the *gyrB* natural promoter was confirmed by colony PCR for the *ori*_R6K_ and PCR amplification of the insertion interface.

### CG mutant library construction

pRB-rham was introduced into *B. cenocepacia* K56-2 via triparental mating (Craig et al. 1989). The exconjugates were selected for by plating onto 500-cm^2^ QTrays (Genetix, X6023; San Jose, CA) containing LB agar with 0.2% rhamnose and the appropriate antibiotics and incubating for 48 h at 37°C. The resulting colonies were robotically picked using a Genetix QPix2 XT colony picker into master plates containing liquid LB medium with 0.1% rhamnose and Tp100 and were incubated overnight. While initial picking and replicating was performed in 96-well plates (Greiner Bio-One, 655185; Monroe, NC), the majority of the library was produced in 384-well microplates (Greiner Bio-One, 781186). The master plates were robotically replicated into secondary plates containing LB and LB 0.1% rhamnose and incubated overnight. Bacterial growth was estimated by measuring OD_600nm_ of the cultures using a BioTek Synergy 2 plate reader equipped with a BioTek Bio-Stack automated plate stacker, and the ratio of growth without and with rhamnose was calculated for each mutant. Transposon mutants showing at least a 50% decrease in OD_600nm_ in the absence of rhamnose in comparison to OD_600nm_ in the presence of rhamnose were manually rescreened for growth in LB with or without 0.1% rhamnose. Mutants showing at least a 50% decrease in OD_600nm_, after 16 h of incubation were stored as glycerol stocks.

### Determination of transposon insertion sites and orientations

Transposon insertion sites were identified either by arbitrary primed PCR (Das et al. 2005; Miller-Williams et al. 2006) or by self-cloning as previously described (Dennis and Zylstra 1998). For each clone, we first attempted arbitrary PCR. A 1-μL aliquot of overnight culture was used directly as the template for an initial low-stringency PCR reaction using a transposon-specific primer and a degenerate arbitrary primer, which amplifies the transposon–genome junction as well as other random stretches of DNA. The products of this reaction were used as the template for a second PCR reaction using an inner transposon-specific primer and a primer identical to the tail of the degenerate primer to preferentially amplify the transposon–genome junction (Table S2). The products were purified using a QIAquick PCR Purification Kit (Qiagen) and sequenced using a third transposon-specific primer. Approximately 20% of the PCR products did not return a usable chromatogram. To determine whether the unsuccessful sequencing was due to the presence of multiple insertions, we subjected these clones to Southern blot experiments. All clones showed only one restricted DNA fragment that hybridized with the transposon-complementary probe, demonstrating that the clones with a failed sequencing reaction harbored a single transposon insertion (data not shown). For these clones, insertions were determined successfully using the self-cloning procedure. DNA was digested using *NotI* or *NdeI*, and Southern blots for the *eGFP* on the end of the transposon were performed using an iBlot Gel Transfer System (Invitrogen, Carlsbad, CA) and DIG High-Prime DNA Labeling Kit (Roche, Indianapolis, IN). The location of the insertion site was determined using nucleotide BLAST against the genome of *B. cenocepacia* J2315 from the *Burkholderia* Genome Database (Winsor et al. 2008). The distance from the insertion to the start site of the downstream open reading frame for insertions into putatively intergenic regions and the start site of the surrounding open reading frame for insertions within putative genes were also calculated.

### Comparisons with essential genes in other bacteria

Essential *E. coli* genes were obtained from PEC (Profiling of *E. coli* Chromosome) (Hashimoto et al. 2005), and from Liberati et al. (2006) for *Pseudomonas aeruginosa*. Orthologs of *B. cenocepacia* J2315 in *E. coli* MG1655 and *P. aeruginosa* PAO1 were found using reciprocal best-hit protein BLAST (Altschul et al. 1990) on the annotated open reading frames with an Expect cutoff of 10. *Escherichia coli* microarray data were obtained from E-MEXP-3461 (Prieto et al. 2011), RNA-seq data from (Yi et al. 2011), and *B. cenocepacia* J2315 microarray data from (Bazzini et al. 2011). The expression levels of essential and nonessential genes were compared using the Mann–Whitney sum-rank test (Lehmann 1998) assuming that the test-statistic *U* is normally distributed given the large sample size. This statistical test assumes neither that the expression is normally distributed across genes nor that there is a linear relationship between the values and the underlying mRNA levels.

### Functional characterization of genes in putatively essential operons

As the rhamnose-inducible promoter will control the expression of all downstream genes in the same transcriptional unit, all downstream genes in the same putative operon were included in our analysis. Genes were included if either OperonDB (confidence level of 50 or more) (Pertea et al. 2009) or DOOR (Database of Prokaryotic Operons) (Dam et al. 2007) placed them in the same putative operon.

### Enhanced sensitivity assay

To calculate rhamnose concentrations that produced 30–60% of wild-type growth, rhamnose dose–response curves of each mutant were run as follows: mutants were grown for 22 h in LB with a rhamnose gradient of 0–0.16%. The resulting OD_600nm_ readings were then fitted to the Hill equation using GraphPad prism (GraphPad Software, Inc., La Jolla, CA). To develop the enhanced sensitivity assay, all the mutants were grown in 96-well-format plates with 200 μL of LB medium containing rhamnose concentrations required to achieve between 30% and 60% of wild-type growth without the addition of antibiotics. Novobiocin (1 μg/mL) or chloramphenicol (2 μg/mL) was added as required. Plates were incubated for 22 h at 37°C with shaking. For each rhamnose concentration, fold reduction was measured as OD_600nm_ without antibiotic/OD_600nm_ with antibiotic, and mutant sensitivity was defined as log_10_ of the fold reduction in growth due to the antibiotic.

## Results

### Building a CG mutant library in *B. cenocepacia* K56-2

We previously defined essential operons as transcriptional units containing at least one essential gene (Cardona et al. 2006). However, the total number of essential genes and their organization into transcriptional units in the large genome of *B. cenocepacia* is unknown. To estimate the number of *B. cenocepacia* essential operons expected to hit to achieve genome coverage, we first analyzed 14 bacterial genomes with experimental data on gene essentiality and compared the number of essential genes versus genome size (Fig. S1). No correlation was found between the size of the essential genome with the overall number of genes with most of the bacterial essential genomes ranging from 300 to 700 genes (Gerdes et al. 2006; Langridge et al. 2009; Christen et al. 2011; Griffin et al. 2011). To estimate the number of operons that may contain essential genes in a model organism, we analyzed the *E. coli* genome in silico, and based on the 306 experimentally confirmed essential genes and a computational estimate of operons (Zhang et al. 2006), we found 180 *E. coli* operons that include one or more essential genes (data not shown). Thus, if *B. cenocepacia* contained 300–700 essential genes as in other bacterial genomes, with a similar distribution to that of *E. coli*, approximately 200–400 essential operons would be expected.

The construction of the *B. cenocepacia* CG mutant library is outlined in Figure 1. The previously developed method for delivering P*rhaB* by transposon mutagenesis and selection by replica plating on LB agar with and without rhamnose (Cardona et al. 2006) was modified to achieve high-throughput levels. After successful transposon mutagenesis and robotic screening in LB liquid media in the presence and absence of rhamnose, transposon mutants with at most 50% growth in the absence of rhamnose were isolated and rescreened before inclusion in the CG library. We reasoned that the permissive 50% cutoff would allow the inclusion of CG mutants with slow growth in the absence of rhamnose due to either low levels of residual expression or remaining essential gene products from growth of the parental culture in rhamnose. This decision also means that the library would include mutants for genes that are important, but not absolutely required for growth. By screening 200,000 transposon mutants with this single condition, 134 CG mutants were isolated and their growth phenotypes confirmed, representing a hit frequency of approximately 1/1500. However, the CG phenotype of 19 of these mutants could not be observed when the original glycerol stocks were further used, leaving 115 CG mutants in the library.

**Figure 2:**
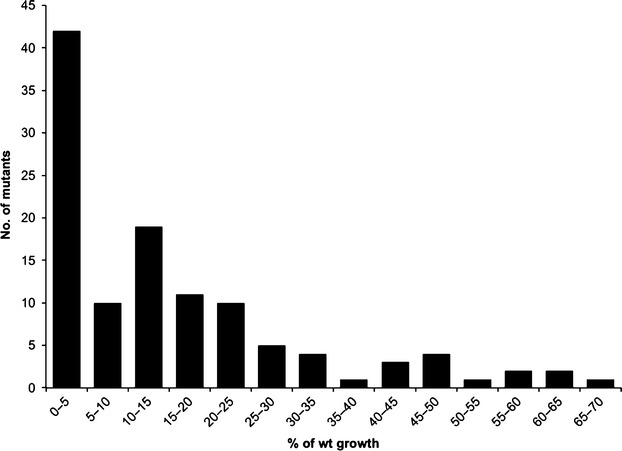
Histogram of growth for 115 CG mutants in the absence of rhamnose. *Burkholderia cenocepacia* K56-2 (wild-type) and CG mutants were grown in LB without rhamnose and OD_600nm_ was measured after 22 h. Percentage of wild-type growth was defined as the growth of CG mutants relative to that of *B. cenocepacia* K56-2. Bars represent the total number of CG mutants with percent of wild-type growth within the range indicated by flanking numbers.

We identified the location and orientation of the delivered P*rhaB* by arbitrary primed PCR (Das et al. 2005; Miller-Williams et al. 2006) or self-cloning (Dennis and Zylstra 1998) and by aligning the obtained DNA sequences with the genome sequence of the clonally related strain J2315 (Holden et al. 2009). All K56-2 DNA sequences could be matched to equivalent genomic regions in J2315. However, we found that a 57-kb duplication on chromosome 1 of J2315 does not exist in the K56-2 genome. Insertion sites in some mutants were mapped to the 3′ end of *lepA1*/*lepA2*, near the middle of the 57-kb duplication on chromosome 1 of J2315. As our system works by usurping the native promoter to control expression of downstream genes, it should be impossible to observe a CG defect from an insertion into only one copy of large identical repeats. After confirming the CG phenotypes in clones that had transposon insertions in the K56-2 putative duplicated region, we verified that the duplication was not present in the genome of K56-2 by PCR amplification of the two duplication-genome interfaces (data not shown). Clones with insertions in this site were the most common ones found with up to 10 clones recovered toward the completion of the screening procedure. Despite the permissive conditions for inclusion into the library the vast majority of the mutants (101 of 115) showed less than 35% of wild-type growth over 22 h in the absence of rhamnose (Fig. 2). Eighty-two mutants in the library showed less than 20% of wild-type growth in the absence of rhamnose, with 42 of these showing less than 5% of wild-type growth over 22 h.

### Functional characterization of essential operons

**Figure 3:**
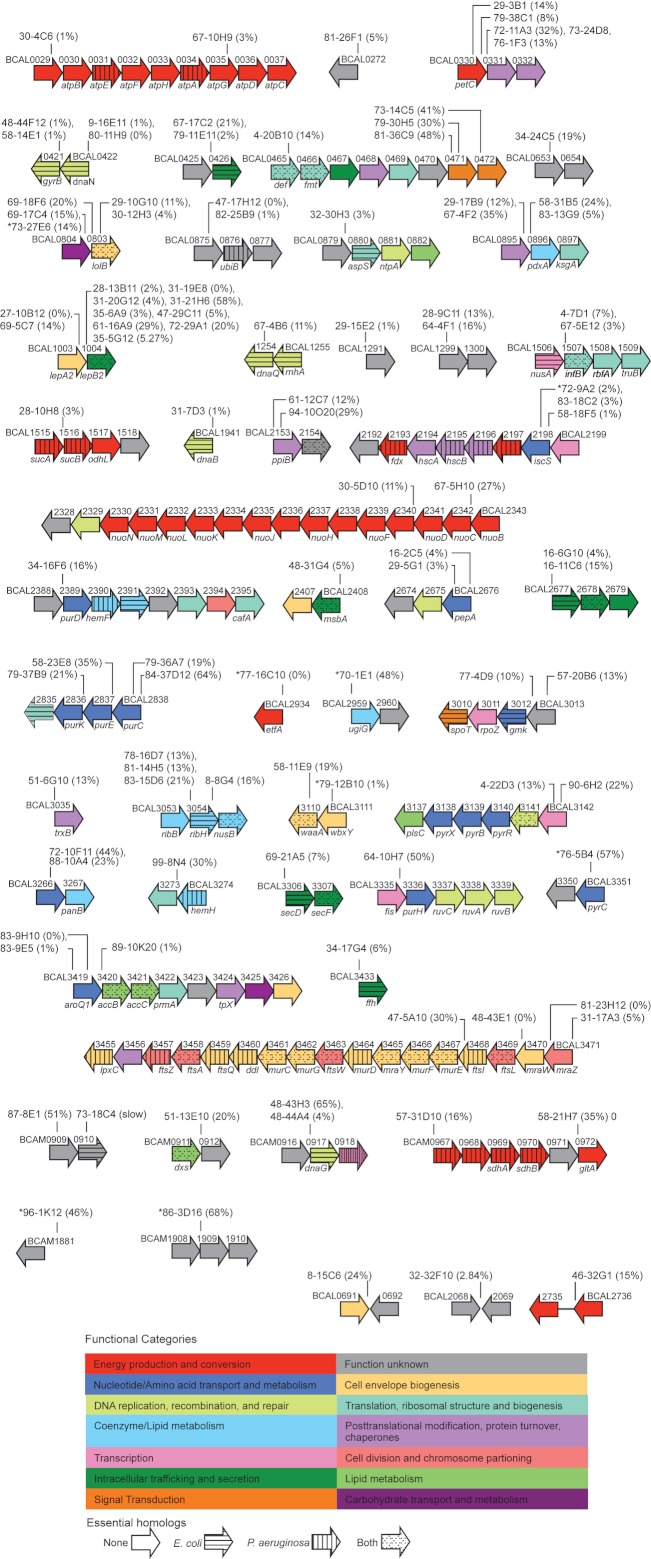
The 50 putative essential operons identified in *Burkholderia cenocepacia* 56-2. Each block represents a putative essential operon, and each arrow represents a gene. Operons include genes downstream from the mutant insertion sites according to OperonDB or DOOR. Genes are ordered according to the locus names of the *B. cenocepacia* J2315 genome. For each mutant, the strain name and the approximate location of the P*rhaB* are indicated by vertical lines. Exact location of insertion sites are listed in Table S1. Mutants where the location of the transposon insertion site could not be determined at the nucleotide level are indicated with an asterisk. The percentage of wild-type growth in the absence of rhamnose is found between brackets beside the strain name. Genes are color coded according to putative function and black patterns indicate essentiality of homologs in *Escherichia coli* (horizontal), *Pseudomonas aeruginosa* (vertical), or both (spots). Three mutant strains, 46-32G1, 8-15C6, and 32-32F10, whose insertion sites and conditional-growth phenotypes are confirmed but no downstream genes appear to be part of the same operon, are shown separately at the end.

**Figure 4:**
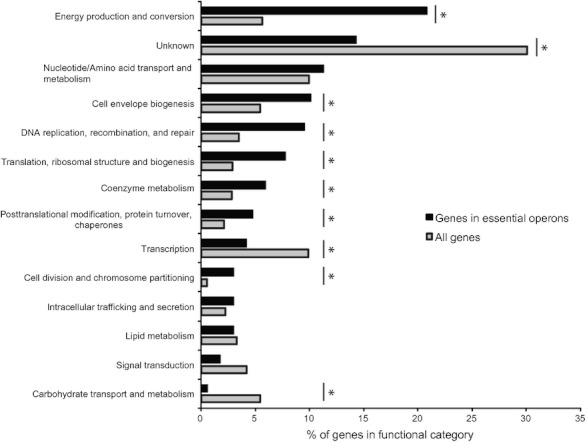
Functional categories of genes in putative essential operons. Putative gene function is based on the GO (Gene Ontology) and COG (Cluster of Orthologous Genes) annotations. For each functional category, Pearson's chi-squared test was used to determine whether the occurrence of the category in the entire genome differs statistically from its occurrence in the putative essential genes identified in this study. A star indicates a *P*-value of less than 0.05.

Of the 115 CG mutants in the library, 106 were successfully sequenced and had insertion sites in the same orientation as interrupted and/or adjacent downstream genes (Fig. 3, Table S1). As P*rhaB* controls the expression of downstream genes in the same operon, insertion sites identified 50 unique putative essential operons (Fig. 3). These operons contained 179 genes, which we organized into functional groups using the COG (Cluster of Orthologous Genes) (Tatusov et al. 2003) and GO (Gene Ontology) (Ashburner et al. 2000) annotations from the *Burkholderia* Genome Database (Winsor et al. 2008). Genes involved in core metabolic functions such as energy production, cell envelope biosynthesis, and DNA replication were overrepresented compared with the entire genome (Fig. 4). Conversely, genes involved in transcription, which are peripheral to growth in the permissive conditions tested, were underrepresented. Similarly, genes involved in carbohydrate metabolism were also underrepresented. This is expected given the range of nutrients available in rich media such as LB. Genes of unknown function were also underrepresented, but still comprised the second largest category after energy production, indicating the large number of uncharacterized genes involved in core *B. cenocepacia* processes.

A BLASTp reciprocal best hit (RBH) revealed 239 *B. cenocepacia* J2315 genes with essential orthologs in *E. coli* (Baba et al. 2008) and 249 with essential orthologs in *P. aeruginosa* PAO1 (Jacobs et al. 2003), of which 131 were essential in both (data not shown). Of the 179 genes found in essential operons in our study, 63 had essential orthologs in either *E. coli* or *P. aeruginosa*, of which 25 were essential in both (Fig. 3, Tables S1 and S3). The identical genomic duplication in *B. cenocepacia* J2315 caused genes in *P. aeruginosa* or *E. coli* that matched to a *B. cenocepacia* J2315 gene in the duplicated region to return two identical “best hits.” As we determined that the aforementioned duplication is not present in the *B. cenocepacia* K56-2 strain used for the experiments, the genes downstream of transposon insertions found in the region by our study were manually matched against *E. coli* and *P. aeruginosa* and added as orthologs to Table S3. The identification of clones with transposon insertions upstream of genes that are orthologous to essential genes related to cell division (*ftsA*, *ftsZ*, *ftsW*, etc.), and peptidoglycan biosynthesis and assembly (*murD*, *mraF*, *mraY*, etc.) shows that our method of large-scale screening for CG mutants does discover operons containing essential genes.

Of the 179 genes found in this study, 117 do not have essential orthologs in *E. coli* or *P. aeruginosa*. Of these, 66 are located in operons with at least one essential ortholog in the aforementioned genomes, for example, BCAL1508 and BCAL1509 (Fig. 3). The remaining 51 genes were organized in 19 operons that did not match to any essential orthologs in either *P. aeruginosa* or *E. coli*. We reasoned that if these genes were essential in *B. cenocepacia*, they might be conserved at least across closely related species. We therefore examined the distribution of these open reading frames across the *Burkholderia* genus using *Burkholderia* Ortholog groups from the *Burkholderia* Genome Database (Winsor et al. 2008). Of the 51 genes, 38 were present in all the sequenced genomes of *Burkholderia* species and *B. cenocepacia* strains (Table S4). This is in agreement with the assumption that gene conservation among *Burkholderia* genomes is related to essentiality (Juhas et al. 2012). However, a few poorly conserved genes that were found in our study also suggest that species or strain-specific requirements for essentiality are also possible. The functions of many of these genes can be inferred from similar genes in other species, but the reasons why they may be required for growth by *B. cenocepacia* K56-2 remains elusive. Seventy-one mutants had insertions inside of putative coding sequences (Figs. 3 and S2). The essentiality of the downstream genes could then be conditional to the absence of the product encoded by the disrupted gene.

### Analyzing the rate at which new essential operons were discovered

**Figure 5:**
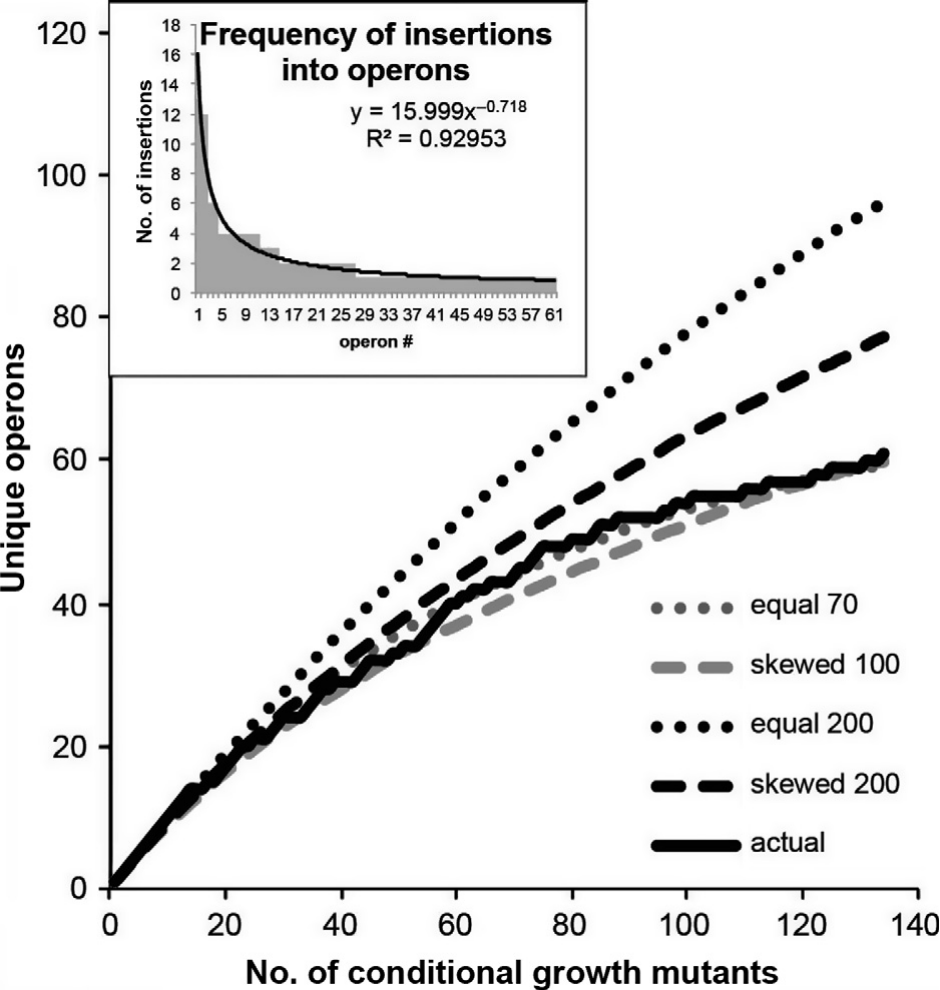
The rate at which new operons were discovered. The rate at which unique operons were discovered experimentally (actual) is compared with four simulations averaging 100 trials of 200, 100, or 70 essential operons, assuming either that every essential operon is equally likely to be detected (equal) or that the observed frequency distribution applies to all essential operons (skewed). The inset shows the frequency at which essential operons were discovered experimentally.

Progress toward the identification of new essential operons was monitored by plotting the number of unique operons discovered against the total number of CG mutants sequenced (Fig. 5). We assumed that as more mutants were sequenced, the proportion of new operons was expected to fall at a rate proportional to the fraction of unique operons already discovered. In addition, the proportion of new operons could also depend on whether all essential operons have the same probability of being discovered. We ran computer simulations of randomly chosen essential operons out of a pool of 200 using either an equal chance of picking every operon or applying the frequency distribution of the experimentally identified essential operons to the entire pool. The actual rate at which new operons were being discovered fell below the theoretical predictions (Fig. 5). When we reduced the theoretical number of essential operons to 70, the simulations matched the experimental results. This means that either the probability of finding certain essential genes with our methodology is unequal or *B. cenocepacia* has a significantly lower number of essential genes than other bacterial genomes. We then analyzed two scenarios that could explain the rate at which new essential operons were being found assuming that *B. cenocepacia* has the same number of essential genes as other bacterial species: limited expression range of the promoter and biased transposon insertion. Alternatively, we analyzed gene redundancy in *B. cenocepacia* as a factor that would render fewer essential genes than expected due to gene duplication.

### Global analysis of essential gene expression levels

It is reasonable to expect that essential genes require strong promoters as they tend to be highly expressed (Dotsch et al. 2010). In previous attempts to modulate essential gene expression via inducible promoters, achieving a CG phenotype has been complicated by the requirement for the chosen system to allow high levels of expression required by essential genes while simultaneously providing tight regulation (Xu et al. 2010). We previously demonstrated that the P*rhaB* is tightly regulated and thus can be used to identify essential genes (Cardona et al. 2006). However, we did not rule out the possibility of missing highly expressed essential genes due to a narrow dynamic range of expression levels driven by P*rhaB*. To examine whether our procedure was excluding highly expressed essential genes, we examined whether global gene expression analysis can show that essential genes are on average more highly expressed than nonessential genes, whether the operons identified by our study showed a similar bias in expression, and finally, whether there was a correlation between frequency at which essential operons were recovered and the level of operon expression.

**Figure 6:**
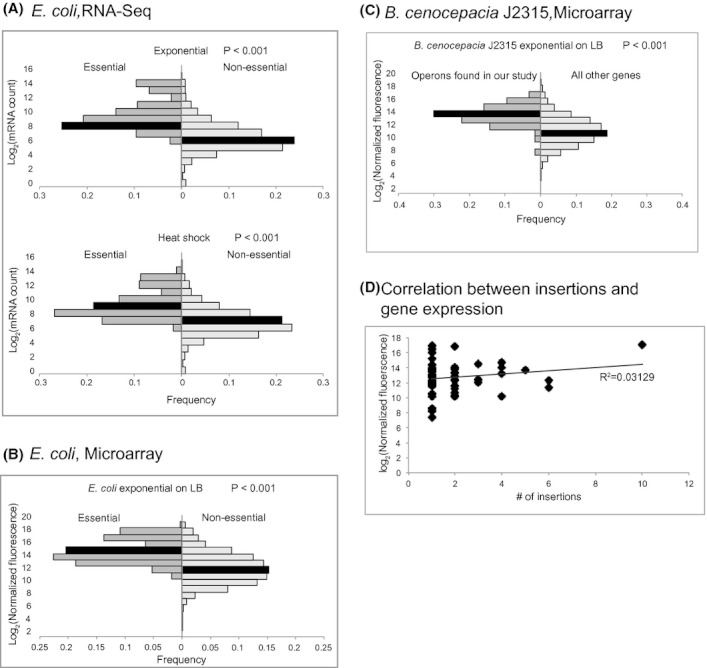
The distribution of gene expression in putatively essential and nonessential genes. The black bars contain the median level of gene expression for each class. The distribution of expression between essential and nonessential genes for each species/condition/methodology was compared using a Mann–Whitney test. Median values, *U* statistic, and number (*n*) of essential and nonessential genes are as follows. (A) Exponentially growing cells (median mRNA transcripts: 364.7 essential, 51.8 nonessential, *U* = 893,584, *P* < 0.001), heat-shock treatment (median mRNA transcripts: 294 essential, 69.3 nonessential, *U* = 786,268, *P* < 0.001) *n*_nonessential_ = 4038, *n*_essential_ = 280; (B) cDNA microarray for exponentially growing cells (median fluorescence: 16,700.09 essential, 3328.65 nonessential, *U* = 701,538, *n*_essential_ = 280, *n*_nonessential_ = 3868, *P* < 0.001); (C) cDNA microarray for *Burkholderia cenocepacia* J2315 (median fluorescence: 10,448.6 operons in library, 1864.7 other genes, *U* = 346,475, *n*_essential_ = 6875, *n*_nonessential_ = 63, *P* < 0.001); (D) There is no correlation between levels of gene expression and the frequency at which insertions into an operon were recovered.

RNA-Seq measures gene expression by sequencing single molecules of mRNA and is thought to provide the most accurate and unbiased absolute quantitation of transcription on a genomic level (Fu et al. 2009). Therefore, we used previously published RNA-Seq data to examine whether essential genes are more likely to be highly expressed in *E. coli* (Prieto et al. 2011). Our analysis showed that *E. coli* essential genes are more highly expressed than nonessential genes in cultures harvested during exponential growth (Fig. 6A), after heat-shock treatment (Fig. 6A), and anaerobic growth (data not shown), with only stationary-phase cells showing no statistically significant difference between essential and nonessential genes (data not shown). As there are no RNA-Seq data available for *B. cenocepacia* J2315 grown in LB, we looked at whether the normalized fluorescence from cDNA microarrays would show similar differences. As microarrays rely on hybridization of labeled cDNA, the fluorescent intensity for any probe depends not only on the number of transcripts, but also on the hybridization efficiency and the possibility of off-target hybridization to other transcripts (Fu et al. 2009). These biases make comparisons of expression levels between different genes based on differences in microarray fluorescence questionable. As we are interested in the difference in expression between essential and nonessential genes in general, these biases should be equally present in both classes of genes. Therefore, we hypothesized that microarrays could substitute for RNA-Seq for our purposes. We confirmed that previously published cDNA microarray data (DeVito et al. 2002) showed similar differences in expression between essential and nonessential *E. coli* genes (Fig. 6B), confirming that there is a bias toward higher levels of expression by essential genes and that this difference can be observed using cDNA microarrays. To determine whether the operons in the CG mutant library were similarly biased toward highly expressed genes, we repeated this analysis using previously published cDNA microarray data for *B. cenocepacia* J2315 grown in LB (Bazzini et al. 2011). As the expression of genes within operons is highly correlated, only the first genes in the identified operons were included in the analysis. There was a significant bias toward high expression of genes from putative essential operons (Fig. 6C). In addition, only 43 genes not represented in our CG mutant library showed higher expression than the most highly expressed operons identified experimentally. Of these, 15 genes in five putative operons have essential orthologs in *E. coli* or *P. aeruginosa*, and all had fluorescence within 2.1-fold of that of the experimentally identified genes. These data suggest the P*rhaB* can drive the levels of expression needed by the vast majority of essential genes.

We next asked if the higher frequency of finding insertions upstream of certain operons was related to hotspots of the Tn*5*-mini transposon. These hotspots have been associated with negatively supercoiled regions (Lodge and Berg 1990) that could be created upstream of highly transcribed genes (Rovinskiy et al. 2012). We then analyzed the relationship between the frequency of recovering CG mutants of certain operons and transcription levels. However, we were unable to find a correlation between a high number of CG mutants recovered for essential operons and high level of expression for those operons (Fig. 6D). Taken together, neither the range of expression levels of the P*rhaB* promoter nor hotspots due to the presence of highly expressed genes can completely explain the observed rate at which new essential operons were discovered.

### Analysis of gene redundancy in *B. cenocepacia*

**Figure 7:**
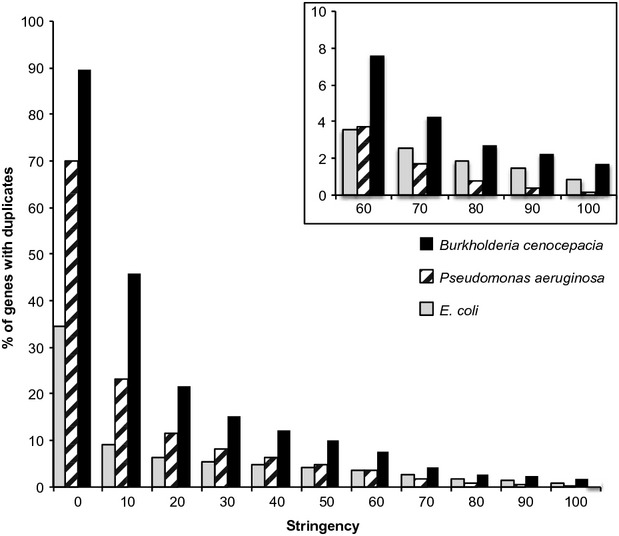
Genetic duplication in *Burkholderia cenocepacia* J2315, *Pseudomonas aeruginosa* PA01, and *Escherichia coli* K12 as a function of DNA sequence similarity. For each strain, the genome was downloaded from the Genome directory of NCBI and a BLAST database was built containing all annotated coding regions. For each gene, similar genes within the same genome were identified using blastn with an expect cutoff of 0.1. Stringency cutoff was defined by percent coverage in the gene alignment and % sequence identity. A stringency cutoff of 40%, for example, means that both percent coverage and sequence identity between two genes are equal or higher than 40%. A gene was included in the same paralogous group when percent coverage and percent identity with at least one member of the group satisfied the stringency cutoff. The inset shows a scaled-up figure of the 60–100 stringency cutoff. Note that paralogous group denotes genes that meet the required conditions for inclusion within the group without any reference to gene history or gene evolution.

To explain the lower than expected rate at which we were discovering essential operons (Fig. 5), we reasoned that a higher number of genes encoding for essential proteins have to be duplicated in comparison with other essential genomes. To address a possible gene-redundancy effect, we first analyzed the criteria for two genes to be considered duplicated. While two exact copies of a gene are undoubtedly duplicates, duplicated genes that diverged after the duplication event may only share a certain DNA sequence similarity over a partially alignable region. To analyze the presence of duplicate genes in *B. cenocepacia*, considering not only identical copies but also duplicate genes that may have further differentiated, we estimated the presence of such genes at different stringency cutoff levels. Similar to other studies of gene duplication (Gu et al. 2002), two parameters were considered for defining stringency: DNA sequence identity and the proportion of alignable sequence over a gene. We compared gene duplications of *B. cenocepacia* with those of *P. aeruginosa* and *E. coli* at all levels of stringency. For example, two genes were considered duplicates with a stringency cutoff of 40 if they shared a DNA sequence identity and percent of alignable sequence equal or higher than 40%. The *B. cenocepacia* genome showed higher gene duplication than the *P. aeruginosa* and *E. coli* genomes at all stringency cutoff levels, but the proportion of duplicates varied greatly with stringency cutoff (Fig. 7). When exact copies were considered (stringency cutoff of 100), approximately 2% and 1% of the genes came out as duplicates for *B. cenocepacia* and *E. coli*, respectively (Fig. 7, inset). This twofold difference was also observed at a stringency cutoff of 60, where the percentage of duplicates increased to 7.6% and 3.5% in *B. cenocepacia* and *E. coli*, respectively. Thus, if the same proportion of duplicated genes observed in whole genomes is observed in essential genomes, then gene redundancy could explain the previously observed lower than expected hit frequency.

### CG mutants demonstrate selective hypersensitivity at low rhamnose concentrations

If a small molecule with antibacterial activity exerts its effect by binding and inhibiting an essential protein, then underexpressing this essential gene should cause cells to become more sensitive to that small molecule (DeVito et al. 2002; Donald et al. 2009). This hypersensitivity should allow growth inhibitors to be matched to their specific molecular targets. We then reasoned that only a CG mutant of the *gyrB* gene should show enhanced sensitivity to the antibiotic novobiocin and other CG mutants should not. Novobiocin exerts its inhibitory action by binding the GyrB subunit of DNA gyrase (Lewis et al. 1996). Using 12 different concentrations of rhamnose and a novobiocin concentration that inhibits 30% of wild-type growth (IC_30_), we established that a *B. cenocepacia gyrB* was hypersensitive at rhamnose concentrations that produced less than 60% of wild-type growth (data not shown). Next, we tested the sensitivity to novobiocin of the CG mutant library at various rhamnose concentrations. We reasoned that while mutants under too little stress may not show any hypersensitivity, severely stressed cells could be generally hypersensitive to growth inhibitors. Reducing growth to 30% or less of wild-type growth caused hypersensitivity and high variability across experiments for most of the CG mutants (data not shown). We then screened 25 randomly chosen mutants at rhamnose concentrations that produced 30–60% of wild-type growth (Fig. S3). Chloramphenicol, which inhibits protein synthesis by binding to the ribosome (Wilson 2011), was used as a negative control at its IC_30_. Only mutants with transposon insertions upstream of *gyrB* were hypersensitive to novobiocin (Fig. S3A), and none of the CG mutants were hypersensitive to chloramphenicol (Fig. S3B).

## Discussion

In this work, we demonstrate that random transposon mutagenesis with an outward-facing promoter P*rhaB* followed by screening for a rhamnose-dependent CG phenotype can identify putative essential operons at a large scale while simultaneously constructing CG mutants. The recovery of genes with essential orthologs in other species known to be involved in essential cellular processes (e.g., *ftsZ*) shows that this methodology is capable of recovering mutants of truly essential genes on a large scale. While secondary site mutations that produce a rhamnose-conditional phenotype cannot be ruled out, we consider this possibility to be unlikely. First, rhamnose is not used as a carbon source in *B. cenocepacia* (data not shown), and it is only known to be part of the O-antigen, a nonessential component of the lipopolysaccharide (Ortega et al. 2005, 2007). Second, we recovered independent transposon mutants that contain the rhamnose promoter controlling the same gene or gene cluster. Finally, independent CG mutants of *gyrB* were constructed showing the same phenotype as recovered by transposon mutagenesis.

If the number of essential genes in *B. cenocepacia* essential genome is not substantially different from what was found in other genomes, our study could identify 20–35% of the *B. cenocepacia* essential operons using a single inducible promoter and single screening condition. A possible explanation for the lower than expected hit frequency could be that the *B. cenocepacia* genome is not uniformly available to the transposon system used. The Tn*5*-mini transposon has been shown to insert into a highly degenerate consensus sequence (Shevchenko et al. 2002) allowing for almost unbiased insertions based on sequence. However, hotspots are common and are associated with local DNA topology with highly transcribed negatively supercoiled regions being more favorable (Lodge and Berg 1990). This suggests that operons with high levels of expression should be more accessible to the transposon, and insertions in these operons should be recovered more frequently. However, among the recovered CG mutants, there was no correlation between the frequency of insertions into an operon and the operon's level of expression (Fig. 6D). Conversely, the presence of DNA regions that may never be targeted by a transposon should be considered. Previously, two independent studies performed in *P. aeruginosa* PAO1 and PA14 used two different transposons, a Tn*5*–based system and a mariner transposon, respectively, to identify putative essential genes (Jacobs et al. 2003; Liberati et al. 2006). Approximately half of the 678 PA14/PAO1 orthologs not hit with Tn*5* were disrupted by the mariner-based transposon in the PA14 library. These 343 genes account for 6.7% of *P. aeruginosa* PAO1/PA14 orthologs, suggesting that only 6.7% of PAO1 genome was missed by the Tn*5* system due to cold spots. Then, a similar distribution of cold spots in *B. cenocepacia* could not account for the rate of essential operon discovery in our study.

The promoters for essential genes differ from those of nonessential genes by having higher levels of expression and lower levels of noise (Silander et al. 2012). In a previous study, genome-wide promoter replacement of essential genes in *Staphylococcus aureus* was attempted by site-directed delivery of tetracycline-inducible promoters with different expression levels. Of 150 essential genes that were targeted with three different promoter variants in more than 400 different constructs, only 64 essential genes were found to have a CG phenotype (Xu et al. 2010). An analysis of the expression levels of the *B. cenocepacia* K56-2 recovered operons showed that the most highly expressed genes in *B. cenocepacia* J2315 were included and that only five predicted essential operons had higher levels of expression than the most highly expressed operons recovered by our study. This suggests that the rhamnose-inducible promoter is capable of driving the expression required by the vast majority of *B. cenocepacia* essential genes when the promoter is delivered randomly and CG mutants are isolated by their conditional phenotype.

The presence of duplicated essential genes in *B. cenocepacia* could lower the hit frequency of our methodology. We predicted that for this effect to account for the lower than expected frequency of finding essential operons, gene duplication in *B. cenocepacia* should be higher than in *E. coli*. Our analysis of *B. cenocepacia* J2315 in comparison with *E. coli* and *P. aeruginosa* showed that gene redundancy in *B. cenocepacia* indeed seems to be twice as high. However, essential genes tend to have fewer paralogs on average than nonessential genes (Deng et al. 2011). Therefore, it is unknown if the observed gene redundancy in *B. cenocepacia* also occurs among essential genes.

In summary, the reasons for the modest success in promoter replacement of essential genes are currently unknown and could be due to effects of the selection conditions or loss of necessary regulatory regions that account for the genomic organization of essential operons in regulons. The frequency at which CG mutants of different essential operons are recovered could also depend on the length of the region into which the transposon can insert and still produce a phenotype (Christen et al. 2011). Promoter replacement of essential genes that occur in clusters or have very short promoters may require high-throughput approaches such as those performed with high-density transposon mutagenesis and next-generation sequencing technology (Gawronski et al. 2009; Langridge et al. 2009).

This study also shows that the *B. cenocepacia* library of CG mutants can be used to screen for the targets of specific growth inhibitors. However, one limitation of this methodology is the inability to detect the target of antibiotics that inhibit growth by forming toxic complexes, rather than by target inactivation. During an antisense RNA study (Xu et al. 2010), an *S. aureus gyrA* mutant was not hypersensitive to ciprofloxacin, an observation we were able to confirm with a *B. cenocepacia* CG mutant of *gyrA* (data not shown). Identifying the target of a small molecule with growth-inhibitory characteristics using the developed assay requires screening the whole library against each compound of interest. As the library is sensitive over a broad range of rhamnose concentrations and sublethal concentrations of novobiocin, we predict that it will be possible to screen pools of CG mutants with broadly similar rhamnose sensitivities. This will allow for the simultaneous screening of many targets against small molecules with growth-inhibitory properties.

## Supporting Information

**Figure S1.** The number of essential genes as a function of genome size. The overall number of essential genes in 14 bacterial genomes is plotted against the total number of genes in the same genome. Data on gene essentiality for all genomes with the exception of data noted with asterisks were collected from Gerdes et al. (2006); gene essentiality of *Caulobacter crescentus, Mycobacterium tuberculosis*, and *Salmonella typhi* were obtained from Christen et al. (2011), Griffin et al. (2011), and Langridge et al. (2009), respectively. Mutants were generated by random or targeted transposon mutagenesis, and mutants were propagated. Mutant lack of survival was considered criteria for defining the interrupted gene as essential. Gray and white squares indicate that the data were obtained from propagating the mutants within a population or clonally, respectively. *Mge*, *Mycoplasma genitalium*; *Hin*, *Haemophilus influenzae* Rd; *Hpy*, *Helicobacter pylori* G27; *Sau*, *Staphylococcus aureus* RN4220; *Mtu*, *M. tuberculosis* H37Rv; *Eco*, *Escherichia coli*; *Sty*, *S. typhi*; *Ccr*, *C. crescentus*; *Bsu*, *Bacillus subtilis*; *PAO1*, *Pseudomonas aeruginosa* PAO1; *PA14*, *P. aeruginosa* PA14.

**Figure S2.** Transposon insertions relative to the start of relevant coding sequence. (A) Histogram of the distance from insertions into putative intergenic regions to the putative start codon of the downstream gene measured in base pairs. (B) Histogram of the distance from insertions inside of putative coding sequences to the start codon of the surrounding gene measured as a percentage of total gene length.

**Figure S3.** Conditional growth mutants show selective hypersensitivity. Mutants were grown in rhamnose concentration gradients estimated to produce more than 30% of wild-type growth and challenged with either novobiocin or chloramphenicol at the IC_30_ of the wild-type. Circles represent CG mutants of the direct target of novobiocin, *gyrB*. Green crosses represent a CG mutant of the electron transfer flavoprotein gene (*etfA)*. Black crosses correspond to nonsensitive mutants. Mutants of *gyrB* show hypersensitivity to novobiocin when grown in rhamnose concentrations producing 30–60% of wild-type growth but not when grown in rhamnose concentrations producing 80–100% of wild-type growth. A CG mutant of *etfA* shows intermediate hypersensitivity. None of the mutants showed hypersensitivity to chloramphenicol. Error bars represent 1 standard deviation calculated from two biological replicates.

**Table S1.** Mutants found in this study.

**Table S2.** Primers.

**Table S3.** Genes found in this study with essential orthologs in *Escherichia coli* or *Pseudomonas aeruginosa*.

**Table S4.** *Burkholderia* species-specific putative essential operons.
