# Supplementary material for: Burkholderia cenocepacia conditional growth mutant library created by random promoter replacement of essential genes
Source: Microbiologyopen. 2013 Feb 7;2(2):243–58. doi: 10.1002/mbo3.71 (PMC3633349; doi:10.1002/mbo3.71)
Supplement: Supplementary file 1 [file mbo30002-0243-SD1.doc]

**Supplementary Tables**

**Supplementary Table 1**. Provided as an Excel file.

This file contains all CG mutants included in the CG mutant library, growth defect, transposon insertion location and downstream genes in putative essential operons.

Reported growth is after incubation for 22h in LB. Some operons called by OperonDB have gaps where a *B. cenocepacia* gene is found between genes, which are adjacent in other genomes.

**Supplementary Table 2**. Primers

| Name | Oligonucleotide sequence, 5’-3’^a^ | Purpose |
| --- | --- | --- |
| 1 | AACACCCGGGATGGTGAGCAAGGGCGAG | Amplification of eGFP |
| 2 | CCGCTCTAGATTACTTGTACAGCTCGTCCA | Amplification of eGFP |
| 34 | CCAGAGGGTACCAAAATTGCTTTGAGAGGCTC | Amplification of *ori* _R6K_ |
| 35 | CTGTTAGGTACCCGT ACTAAGCTCTCATGTTT | Amplification of *ori* _R6K_ |
| 178 | GGCCACGCGTCGACTAGTCAGNNNNNNNNNNACGCC | Reverse primer for 1^st^ round of arbitrary PCR |
| 179 | GGCCACGCGTCGACTAGTCAG | Reverse primer for 2^nd^ round of arbitrary PCR |
| 187 | ACATCACCACAATTCAGCAA | Forward outer transposon specific primer |
| 188 | ACCCAGTCCGCCCTGAGCAA | Forward inner transposon specific primer |
| 189 | CCCTGAGCAAAGACCCCAAC | Sequencing insertion site |
| 06 | AATTAACATATGATGAGTGAACAGCACAATTCGC | Amplification of 300bp 5’ end of *gyrB* |
| 207 | ATATAATCTAGACTTCGGCTCGTGCTTGTCG | Amplification of 300bp 5’ end of *gyrB* |
| 249 | CCCGACACCTTGTAGCTGTTCTG | Reverse external primer for confirming *gyrB* insertion |

^a^ Restriction sites are underlined

***Supplementary Table 3****. Genes found in this study with essential orthologs in E. coli or* P. aeruginosa

| **Locus** | ***P.aeruginosa* ortholog** | ***E.coli* ortholog** | **Function** | **Essential in^a^** | |
| --- | --- | --- | --- | --- | --- |
|  |  |  |  | ***P. aeruginosa*** | ***E. coli*** |
| BCAL0031 | PA5559 | b3737 | *atpE*, ATP synthase | E | N |
| BCAL0034 | PA5556 | b3734 | *atpA*, ATP synthase | E | N |
| BCAL0421 | PA0004 | b3699 | *gyrB*, DNA gyrase | N | E |
| BCAL0422 | PA0002 | b3701 | *dnaN*, DNA polymerase III, beta subunit | N | E |
| BCAL0426 | PA5568 | b3705 | *yidC*, membrane protein insertase | N | E |
| BCAL0465 | PA0019 | b3287 | *def*, peptide deformylase | E | E |
| BCAL0466 | PA0018 | b3288 | *fmt*, Aminoacyl tRNA synthetases | E | E |
| BCAL0803 | PA4668 | b1209 | *lolB*, localization of lipoproteins | E | E |
| BCAL0876 | PA5065 | b3835 | *ubiB*, 2-octaprenylphenol hydroxylase | E | N |
| BCAL0880 | PA0963 | b1866 | *aspS*, aspartyl-tRNA synthetase | N | E |
| BCAL1004 | PA0768 | b2568 | *lepB2*, signal peptidase I 2 | N | E |
| BCAL1254 | PA1816 | b0215 | *dnaQ*, DNA polymerase III epsilon subunit | E | N |
| BCAL1255 | PA1815 | b0214 | *rnhA*, degrades RNA of DNA-RNA hybrids | E | N |
| BCAL1506 | PA4745 | b3169 | *nusA*, transcription termination | N | E |
| BCAL1507 | PA4744 | b3168 | *infB*, Translation inititation | E | E |
| BCAL1515 | PA1585 | b0726 | *sucA*, oxoglutarate dehydrogenase | E | N |
| BCAL1516 | PA1586 | b0727 | *sucB*, 2-oxoglutarate dehydrogenase | E | N |
| BCAL1941 | PA4931 | b4052 | *dnaB*, replicative DNA helicase | N | E |
| BCAL2154 | PA1792 | b0524 | *lpxH*, Lipid A biosynthesis | E | E |
| BCAL2193 | PA3809 | b2525 | *fdx*, [2Fe-2S] ferredoxin | E | N |
| BCAL2195 | PA3811 | b2527 | *hscB*, chaperone specific for IscU | E | N |
| BCAL2196 | PA3812 | b2528 | *iscA*, FeS cluster assembly | E | N |
| BCAL2197 | PA3813 | b2529 | *iscU*, FeS cluster assembly | E | N |
| BCAL2390 | PA0024 | b2436 | *hemF*, coproporphyrinogen III oxidase | E | N |
| BCAL2391 | PA4006 | b0639 | *nadD*, NAD biosynthesis | N | E |
| BCAL2408 | PA4997 | b0914 | *msbA*, ATP-binding transport protein | E | E |
| BCAL2677 | PA3828 | b4261 | *lptF*, lipopolysaccharide export permease | N | E |
| BCAL2678 | PA3827 | b4262 | *lptG*, lipopolysaccharide export permease | E | E |
| BCAL2835 | PA0022 | None | conserved hypothetical protein | E | NA |
| BCAL2959 | PA3171 | b2232 | *ubiG*, 3-demethylubiquinone-93-methyltransferase/  2-octaprenyl-6-hydroxy phenol methylase | E | N |
| BCAL3010 | PA5338 | b3650 | *spoT*, (p)ppGpp synthetase | N | E |
| BCAL3012 | PA5336 | b3648 | *gmk*, guanylate kinase | N | E |
| BCAL3054 | PA4053 | b0415 | *ribE*, riboflavin synthase | N | E |
| BCAL3055 | PA4052 | b0416 | *nusB*, transcription antitermination | E | E |
| BCAL3110 | PA4988 | b3633 | *waaA*, KDO transferase | E | E |
| BCAL3141 | PA0404 | b2949 | *yqgF*, predicted Holliday junction resolvase | E | E |
| BCAL3274 | PA4655 | b0475 | *hemH*, ferrochelatase | E | E |
| BCAL3306 | PA3821 | b0408 | *secD*, protein translocase auxillary subunit | N | E |
| BCAL3307 | PA3820 | b0409 | *secF*, protein translocase auxillary subunit | E | E |
| BCAL3420 | PA4847 | b3255 | *accB*, acetyl CoA carboxylase | E | E |
| BCAL3421 | PA4848 | b3256 | *accC*, acetyl-CoA carboxylase | E | E |
| BCAL3433 | PA3746 | b2610 | *ffh*, Signal Recognition Particle | N | E |
| BCAL3455 | PA4406 | b0096 | *lpxC*, lipidA biosynthesis | N | E |
| BCAL3457 | PA4407 | b0095 | *ftsZ*, cell division | N | E |
| BCAL3458 | PA4408 | b0094 | *ftsA*, recruitment of FtsK to Z ring | E | E |
| BCAL3459 | PA4409 | b0093 | *ftsQ*, ingrowth of wall at septum | N | E |
| BCAL3461 | PA4411 | b0091 | *murC*, UDP-N-acetylmuramate:L-alanine ligase | E | E |
| BCAL3462 | PA4412 | b0090 | *murG*, peptidoglycan biosynthesis | E | E |
| BCAL3463 | PA4413 | b0089 | *ftsW*, stabilizing FstZ ring during cell division | E | E |
| BCAL3464 | PA4414 | b0088 | *murD*, peptidoglycan biosynthesis | N | E |
| BCAL3465 | PA4415 | b0087 | *mraY*, peptidoglycan biosynthesis | E | E |
| BCAL3466 | PA4416 | b0086 | *murF*, peptidoglycan biosynthesis | E | E |
| BCAL3467 | PA4417 | b0085 | *murE*, peptidoglycan biosynthesis | E | E |
| BCAL3468 | PA2272 | b0084 | *ftsI*, peptidoglycan biosynthesis | N | E |
| BCAL3469 | PA4419 | b0083 | *ftsL*, ingrowth of wall at septum | E | E |
| BCAM0910 | PA4043 | b0421 | putative geranyltranstransferase | N | E |
| BCAM0911 | PA4044 | b0420 | *dxs*, 1-deoxyxylulose-5-phosphate synthase | E | E |
| BCAM0917 | PA0577 | b3066 | *dnaG*, DNA primase | N | E |
| BCAM0918 | PA0576 | b3067 | *rpoD*, RNA polymerase, sigma 70 | E | E |
| BCAM0967 | PA1581 | b0721 | *sdhC*, succinate dehydrogenase | E | N |
| BCAM0968 | PA1582 | b0722 | *sdhD*, succinate dehydrogenase | E | N |
| BCAM0969 | PA1583 | b0723 | *sdhA*, succinate dehydrogenase | E | N |
| BCAM0970 | PA1584 | b0724 | *sdhB*, succinate dehydrogenase | E | N |

^a^ E – Essential, N – Nonessential, NA not applicable due to lack of a homolog

**Supplementary Table 4**. Burkholderia-species specific putative essential operons

|  |  |  | Contains at least one gene in the same ortholog group | | | | | | | | | | | |
| --- | --- | --- | --- | --- | --- | --- | --- | --- | --- | --- | --- | --- | --- | --- |
| Locus | Gene name or function | Mutants with insertion site at the beginning of or immediately upstream from the locus | Bcc AU 1054 | Bcc H12424 | Bcc MC0-3 | Bam | Bla | Bma | Bmu | Bph | Bps | Bth | Bvi | Bxe |
| BCAL0272 | Putative lipoprotein | 81-26F1 | • | • | • | • |  |  | • | • |  |  | • | • |
| BCAL0330 | *petC* | 29-3B1, 73-24D8, 72-11A3, 73-24D8, 76-1F3 | • | • | • | • | • | • | • | • | • | • | • | • |
| BCAL0331 | Putative stringent starvation protein A |  | • | • | • | • | • | • | • | • | • | • | • | • |
| BCAL0332 | *ClpXP* |  | • | • | • | • | • | • | • | • | • | • | • | • |
| BCAL0653 |  | 34-24C5 | • | • | • |  | • | • |  |  | • | • |  |  |
| BCAL0654 |  |  | • | • | • |  |  |  |  |  |  |  |  |  |
| BCAL0691 | putative cytidylyltransferase | 8-15C6 | • | • | • | • | • | • | • | • | • | • | • | • |
| BCAL0895 | Putative *surA* | 29-17B9, 67-4F2 | • | • | • | • | • | • | • | • | • | • | • | • |
| BCAL0896 | *pdxA* | 58-31B5, 83-13G9 | • | • | • | • |  | • | • | • | • | • | • | • |
| BCAL0897 | *ksgA* |  | • | • | • | • |  | • | • | • | • | • | • | • |
| BCAL1291 |  | 29-15E2 |  |  |  |  |  |  |  |  |  |  |  |  |
| BCAL1299 |  | 28-9C11, 64-4F1 |  |  |  |  |  |  |  |  |  |  |  |  |
| BCAL1300 |  |  | • |  |  | • |  |  |  |  |  |  |  |  |
| BCAL2068 |  | 32-32F10 | • | • | • | • | • | • | • | • | • | • | • | • |
| BCAL2343 | *nuoB* | 67-5H10 | • | • | • | • | • | • | • | • | • | • | • | • |
| BCAL2342 | *nuoC* |  | • | • | • | • | • | • | • | • | • | • | • | • |
| BCAL2341 | *nuoD* |  | • | • | • | • | • | • | • | • | • | • | • | • |
| BCAL2340 | Putative *nuoE* | 30-5D10 | • | • | • | • | • | • | • | • | • | • | • | • |
| BCAL2339 | *nuoF* |  | • | • | • | • | • | • | • | • | • | • | • | • |
| BCAL2338 | Putative *nuoG* |  | • | • | • | • | • | • | • | • | • | • | • | • |
| BCAL2337 | *nuoH* |  | • | • | • | • | • | • | • | • | • | • | • | • |
| BCAL2336 | Putative *nuoI* |  | • | • | • | • | • | • | • | • | • | • | • | • |
| BCAL2335 | *nuoJ* |  | • | • | • | • | • | • | • | • | • | • | • | • |
| BCAL2334 | *nuoK* |  | • | • | • | • | • | • | • | • | • | • | • | • |
| BCAL2333 | *nuoL* |  | • | • | • | • | • | • | • | • | • | • | • | • |
| BCAL2332 | *nuoM* |  | • | • | • | • | • | • | • | • | • | • | • | • |
| BCAL2331 | *nuoN* |  | • | • | • | • | • | • | • | • | • | • | • | • |
| BCAL2330 |  |  | • | • | • | • | • | • | • | • | • | • | • | • |
| BCAL2329 | NUDIX hydrolase |  | • | • | • | • | • | • | • | • | • | • | • | • |
| BCAL2328 |  |  | • | • | • | • | • | • | • | • | • | • | • | • |
| BCAL2676 | *pepA* | 16-2C5 | • | • | • | • | • | • | • | • | • | • | • | • |
| BCAL2675 | DNA polymerase III chi subunit |  | • | • | • | • | • | • | • | • | • | • | • | • |
| BCAL2674 |  |  | • | • | • | • | • | • | • | • | • | • | • | • |
| BCAL2736 | isocitrate dehydrogenase | 46-32G1 | • | • | • | • | • | • | • | • | • | • | • | • |
| BCAL2934 | *etfA* | 77-16C10 | • | • | • | • | • | • | • | • | • | • | • | • |
| BCAL2959 | *ugiG* | 70-1E1 | • | • | • | • | • | • | • | • | • | • | • | • |
| BCAL2960 |  |  | • | • | • | • | • | • | • | • | • | • | • | • |
| BCAL3035 | *trxB* | 51-6G10 | • | • | • | • | • | • | • | • | • | • | • | • |
| BCAL3266 | Putative deoxynucleotide kinase | 72-10F11, 88-10A4 | • | • | • | • | • | • | • | • | • | • | • | • |
| BCAL2367 | *panB* |  | • | • | • | • | • | • | • | • | • | • | • | • |
| BCAL3335 | *fis* | 64-10H7 | • | • | • | • | • | • | • | • | • | • | • | • |
| BCAL3336 | *purH* |  | • | • | • | • | • | • | • | • | • | • | • | • |
| BCAL3337 | *ruvC* |  | • | • | • | • | • | • | • | • | • | • | • | • |
| BCAL3338 | *ruvA* |  | • | • | • | • | • | • | • | • | • | • | • | • |
| BCAL3339 | *ruvB* |  | • | • | • | • | • | • | • | • | • | • | • | • |
| BCAL3351 | *pyrC* | 76-5B4 | • | • | • | • | • | • | • | • | • | • | • | • |
| BCAL3350 |  |  | • | • | • |  |  | • | • | • | • | • | • | • |
| BCAM1881 |  | 96-1K12 |  |  |  |  |  |  |  |  |  |  |  |  |
| BCAM1908 |  | 86-3D16 |  |  |  |  |  |  |  |  |  |  |  |  |
| BCAM1909 |  |  |  |  |  |  |  |  |  |  |  |  |  |  |
| BCAM1910 |  |  |  |  |  |  |  |  |  |  |  |  |  |  |

Bcc, *B. cenocepacia;* Bam, *B. ambifaria;* Bla, *B. lata*; Bma, *B. mallei*; Bmu, *B. multivorans;* Bph, *B. phymatum;* Bps, *B. pseudomallei;* Bth, *B. thailandensis;* Bvi, *B. vietnamiensis;* Bxe, *B. xenovorans.*

**Supplementary Figures**


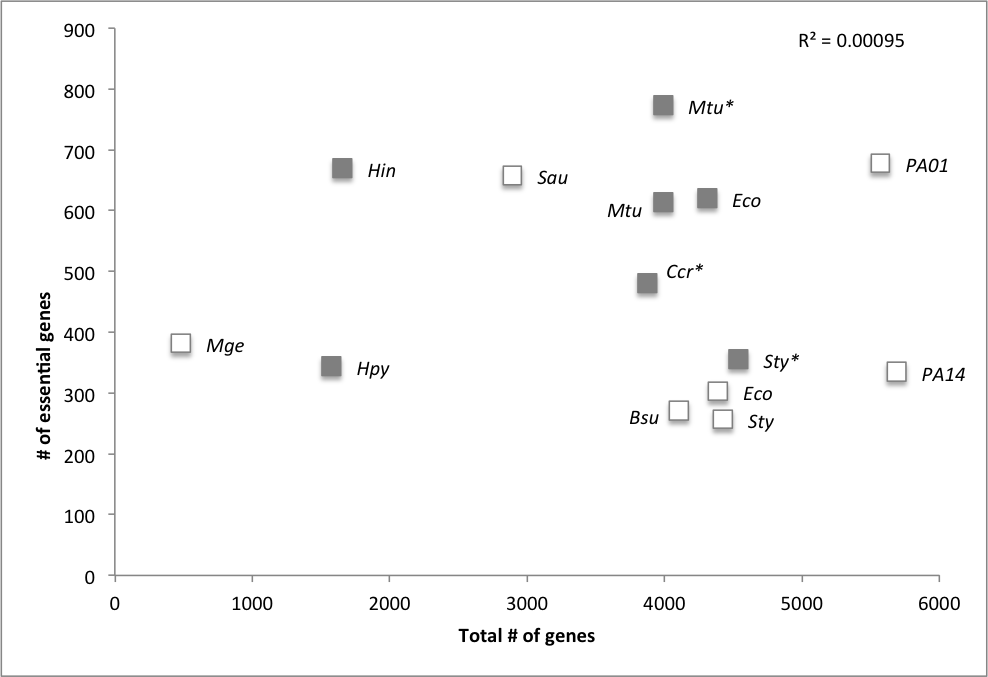


**Supplementary Fig. 1**. **The number of essential genes as a function of genome size.** The overall number of essential genes in 14 bacterial genomes is plotted against the total number of genes in the same genome. Data on gene essentiality for all genomes with the exception of data noted with asterisks was collected from Gerdes *et al.*, 2006. Gene essentiality of *C. crescentus*, *M. tuberculosis* and *S. Typhi* were obtained from Christen *et al*., 2011, Griffin *et al*., 2011 and Landgridge *et al*., 2009, respectively. Mutants were generated by random or targeted transposon mutagenesis, and mutants were propagated. Mutant lack of survival was considered criteria for defining the interrupted gene as essential. Grey and white squares indicate that the data was obtained from propagating the mutants within a population or clonally, respectively. *Mge*, *Mycoplasma genitalium*; *Hin*, *Haemophilus influenzae* Rd; *Hpy*, *Helicobacter* *pylori* G27; *Sau*, *Staphylococcus aureus* RN4220; *Mtu*, *Mycobacterium tuberculosis* H37Rv; *Eco*, *Escherichia coli*; *Sty*, *Salmonella Typhi*; *Ccr*, *Caulobacter crescentus; Bsu, Bacillus subtilis; PAO1*, *Pseudomonas aeruginosa* PAO1; *PA14*, *P. aeruginosa* PA14.

**Supplementary Fig. 2**


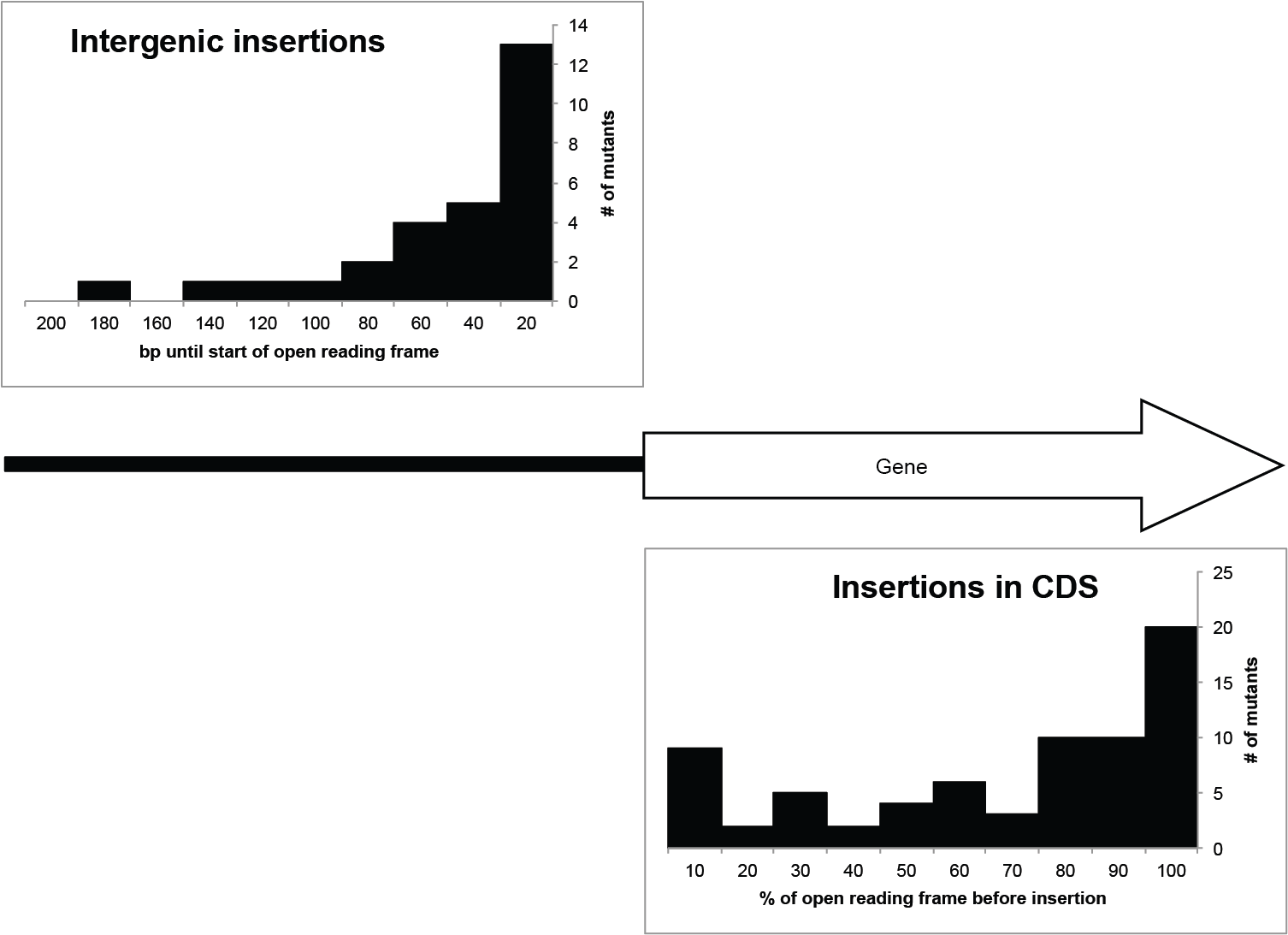


**Supplementary Fig. 2**. **Transposon insertions relative to the start of relevant coding sequence.** A) Histogram of the distance from insertions into putative intergenic regions to the putative start codon of the downstream gene measured in base pairs. B) Histogram of the distance from insertions inside of putative coding sequences to the start codon of the surrounding gene measured as a percentage of total gene length.

**Supplementary Fig. 3**


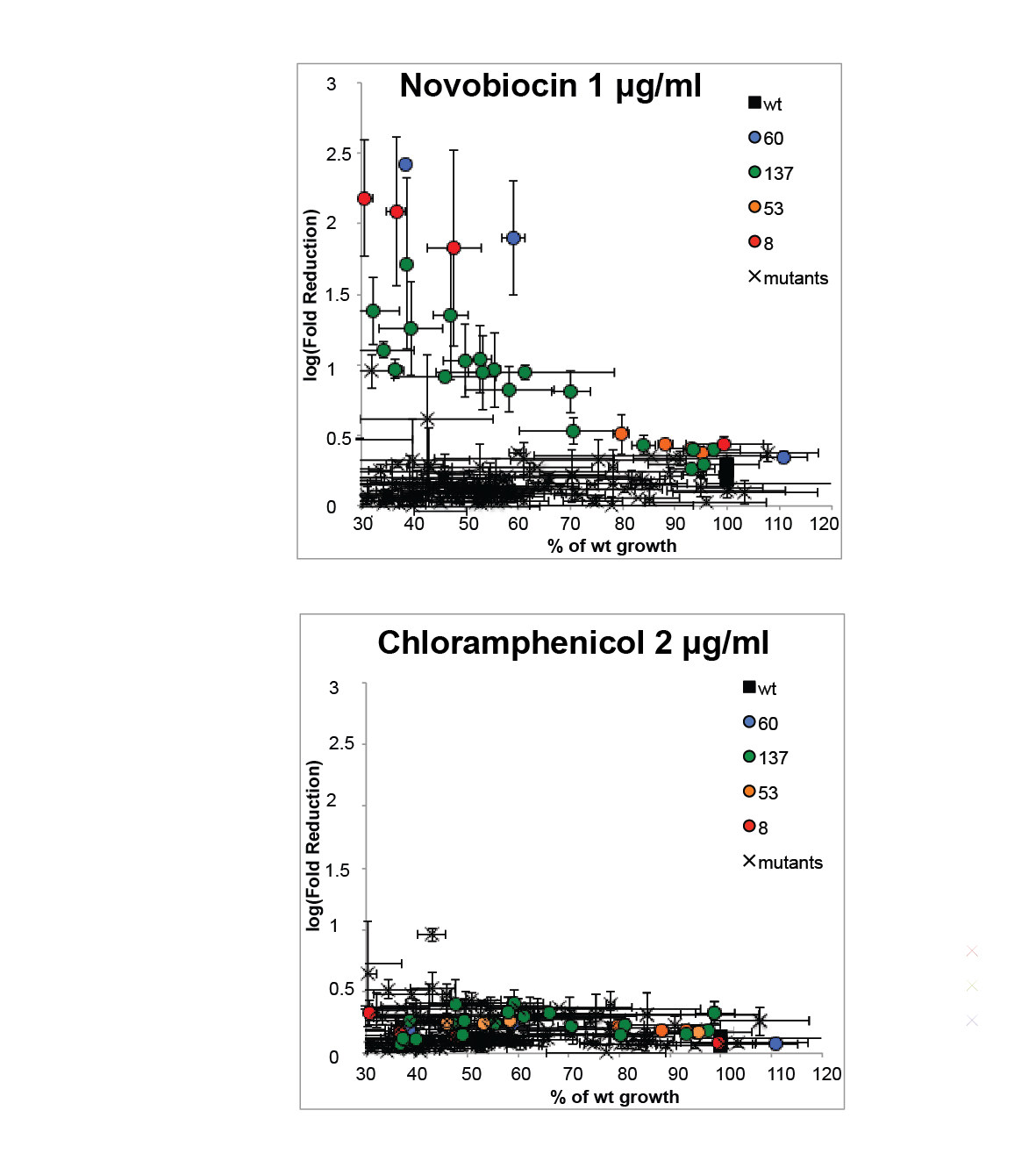


**Supplementary Fig. 3. Conditional growth mutants show selective hypersensitivity.** Mutants were grown in rhamnose concentration gradients estimated to produce more than 30% of wild type growth and challenged with either novobiocin or chloramphenicol at the IC_30_ of the wild type. Circles represent CG mutants of the direct target of novobiocin, gyrB. Green crosses represent a CG mutant of the electron transfer flavoprotein gene (*etfA).* Black crosses correspond to non-sensitive mutants. Mutants of *gyrB* show hypersensitivity to novobiocin when grown in rhamnose concentrations producing 30 to 60% of wild type growth but not when grown in rhamnose concentrations producing 80 to 100% of wild type growth. A CG mutant of *etfA* shows intermediate hypersensitivity. None of the mutants showed hypersensitivity to chloramphenicol. Error bars represent 1 standard deviation calculated from 2 biological replicates.
